# Preventing Laryngeal Nerve Palsy During Thyroidectomies: A Non-systematic Review of the Surgical Anatomy Literature

**DOI:** 10.7759/cureus.98801

**Published:** 2025-12-09

**Authors:** Shahzeb Sheikh, James Moor

**Affiliations:** 1 Acute Medicine, Nottingham University Hospitals NHS Trust, Nottingham, GBR; 2 Otolaryngology - Head and Neck Surgery, Leeds University Hospitals NHS Trust, Leeds, GBR

**Keywords:** dysphonia, head and neck cancer, speech and language, thyroid, voice

## Abstract

This non-systematic review explored recurrent laryngeal nerve (RLN) injury mechanisms, anatomical landmarks, and extralaryngeal branching (ELB) variations during thyroidectomy. A PubMed and Medline search identified relevant studies, including systematic reviews and meta-analyses. Key risk factors for RLN palsy include repeat surgeries, extent of resection, and malignancy. Injury mechanisms involve traction, compression, and thermal damage. The Zuckerkandl tubercle and Berry’s ligament show consistent RLN relationships with greater clinical utility than the inferior thyroid artery. ELB, especially bifurcation, increases RLN injury risk. Understanding RLN anatomy and variations is crucial to minimizing injury. Further studies are needed to assess the clinical significance of these anatomical landmarks and variations to guide safer surgeries.

## Introduction and background

Thyroidectomy is a surgical procedure which involves removing all or part of the thyroid gland and is indicated in a variety of conditions, such as goitre, hyperthyroidism, suspicious nodules and malignancy [1]. Thyroidectomy is regarded to be a relatively safe and cost-effective procedure; however, a number of risks are posed to vital structures around the thyroid gland, most importantly the recurrent laryngeal nerve (RLN) [2,3]. The RLN is a branch of the vagus nerve and supplies all the muscles of the larynx except for the cricothyroid [4]. The laryngeal muscles are vital to permit normal phonation, swallowing and breathing [5]. The RLN is at risk during thyroidectomy procedures due to its close anatomical relationship with the thyroid gland, and injury can cause vocal cord palsy (also described in the literature as recurrent laryngeal nerve palsy (RLNP)). Consequently, symptoms include hoarseness, difficult or abnormal swallowing and breathing difficulties [6].

The RLN is known to have a high number of anatomical variations, which makes identification difficult and the risk of injury higher; hence, RLNP is associated with a high number of malpractice claims [7]. It is vital that thyroid surgeons are aware and cognisant of the anatomical risk factors and common mechanisms of injury. Furthermore, anatomical landmarks and variants of the RLN need to be examined while subsequently implementing efficacious methods to prevent damage. The aim of this non-systematic review is to evaluate the mechanisms of injury to the RLN, the anatomical landmarks and variations.

## Review

Materials and methods

A literature search was carried out using PubMed and Medline to identify relevant papers using Boolean operators with key terms including “recurrent laryngeal nerve variations” and “anatomical landmarks”. Multiple combinations of keywords, including thyroidectomy, hemithyroidectomy, total thyroidectomy, thyroid surgery, Berry’s ligament, Zuckerkandl tubercle and Boolean operators, were used in combination specific to the type of anatomical landmark or method of nerve injury prevention being investigated. Inclusion criteria were added, including journal articles, systematic reviews and meta-analyses. For example, “tracheoesophageal groove” AND “recurrent laryngeal nerve AND thyroidectomy” yielded 31 results in PubMed. The abstracts were analysed for relevance in the top 40 results for each section of the review, and selected papers were investigated further. Furthermore, various meta-analyses and systematic reviews were analysed for relevant citations to be investigated further. Any articles which included dissertations, letters to authors, conference literature and dissertations were excluded.

Results

Risk Factors and Mechanisms of Injury

As would be expected, numerous studies have shown that 3-5% of patients experience RLNP after thyroidectomy [8-10]. One meta-analysis published rates of temporary RLNP postoperatively ranging from 1.4% to 38% [11]. Permanent RLN palsy rates range from 0.2% to 18.6% [11].

Several risk factors have been known to influence the rates of RLNP. Repeat thyroid operations have shown an increased risk of RLNP, with a relative risk of 3.1 in revision goitre surgery compared to primary surgery [12]. Formation of adhesions and scarring causes difficulties in identifying and dissecting the nerve [12,13]. Additionally, a preoperative diagnosis of thyroid malignancy has been shown to increase the risk of RLNP after thyroidectomy, with around 15.4% of patients compared to 4.8% in patients with benign thyroid disease, while another study showed that a preoperative diagnosis of thyroid cancer imparts a relative risk of RLNP of 5.4 compared to benign diagnoses [14,15]. Invasion of the RLN and surrounding tissue by the extra-thyroidal extension of the tumour causes difficulty in dissection of the nerve due to distortion of local anatomy and hence increases the risk of intraoperative damage [16]. Furthermore, the type and extent of thyroidectomy impact the extent of RLN damage since a larger resection of the thyroid gland confers a greater risk of RLNP [17]. Another study highlighted that RLNP is higher in groups of patients undergoing total thyroidectomy at 2% compared to 0.2% in patients with subtotal/hemithyroid surgery [17].

Various mechanisms are known to cause injury to the RLN during a thyroidectomy. Traction on the RLN when distracting the thyroid gland medially can cause stretching of the RLN due to its relatively fixed entry point to the larynx posterior to Berry’s ligament (BL) [17]. While the nerve as it ascends the neck is relatively mobile, the process of rotation of the thyroid lobe to expose the tracheoesophageal groove (TOG) can cause the RLN to be stretched [18]. Furthermore, stretching can occur due to intraoperative compression from a dense fibrous adhesion band or by being stretched as a multinodular goitre has expanded medial and posterior to the nerve, hence causing the nerve to lie in a highly unusual location on the anterolateral aspect of an enlarged thyroid, thus increasing the likelihood of injury [18]. Around 71% of injuries to the RLN were found to have been caused by traction injuries; however, the injury is usually mild, and RLNP is temporary [19]. Other modes of mechanical trauma include application of pressure directly on the RLN due to compression or by clamping the nerve inadvertently [20]. Constriction injuries occur via RLN entrapment within a structure or ligaclip used to control small vessel bleeding or a constricting band of tissue during dissection of the nerve [20]. Thermal injuries are induced via a high-energy instrument such as monopolar or bipolar diathermy, or more novel surgical devices, including the Harmonic Scalpel or Ligasure. Subsequent RLNP due to excessive heat exposure is associated with longer recovery times and a higher rate of permanent palsy at around 28% [20]. Transection injuries are the most problematic and cause complete RLNP due to total severing of the axons and sheath within the nerve, but are also the rarest form of injury [19].

Anatomical Landmarks

Several anatomical landmarks can be used to aid in the identification of the RLN during thyroidectomy [21]. Substantial variation exists in the position of the RLN in relation to these, altering their effectiveness as useful, consistent landmarks. Anatomical variations, such as extralaryngeal branching (ELB), can increase the risk of RLNP for inexperienced thyroid surgeons; hence, it is essential that surgeons have an awareness of them [22].

Tracheoesophageal Groove (TOG)

The TOG is formed by the trachea and oesophagus abutting together and forming a defined indentation for the RLN to reside either within or outside [23]. The RLN position can vary in relation to the TOG [23].

Nine papers were identified that investigated the relationship between the RLN and TOG, one of which was a meta-analysis. This compared 23 studies and found a statistically significant difference between the prevalence of the RLN within the TOG in 63.7% of cases and outside in 36.3% of cases (p < 0.001) [23]. There were no statistically significant differences according to subgroup analysis, such as type of study, side being investigated and geographical origin of subjects. A cadaveric study by Al-Salihi and Dabbagh showed that out of 212 RLNs investigated, 176 nerves were found to be in the TOG, around 83% in total [23]. Furthermore, according to Gupta et al., almost 70% of RLNs were found in the TOG when 162 RLNs were dissected intraoperatively in 140 patients undergoing thyroidectomies [24]. Concurrently, Chang et al. used the TOG as a landmark in all of their endoscopic thyroidectomies in 120 patients and subsequently identified the RLN easily within 15 minutes [25]. They considered the TOG to be a reliable anatomical landmark, and simple palpation can provide valuable information as to the location of the nerve in intraoperative surgery [24,25]. However, the latter two studies looked at significantly more females compared to males, with a ratio of almost 12:1, indicating potential selection bias [24,25].

**Figure 1:**
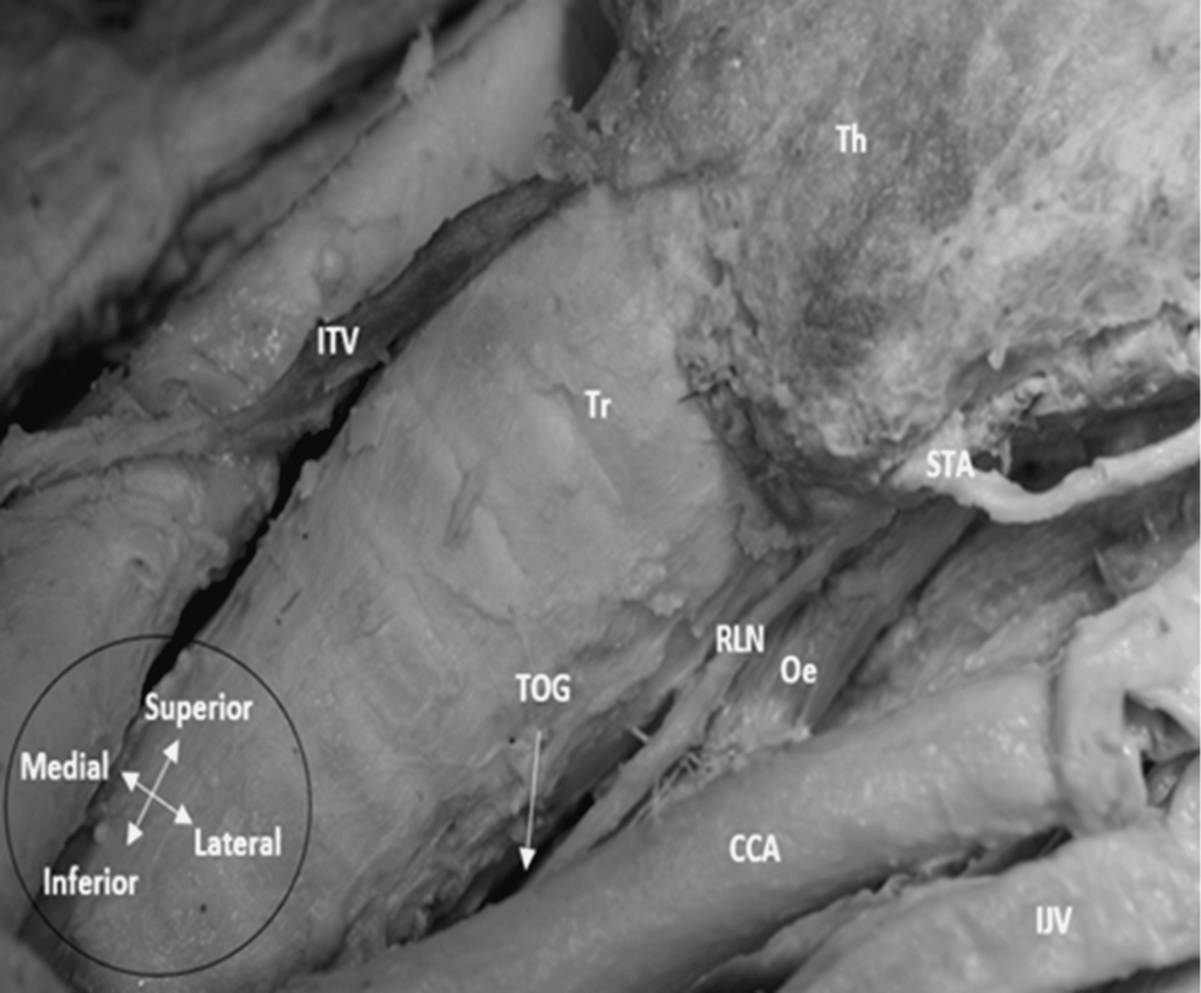
Left recurrent laryngeal nerve inside the tracheoesophageal groove on a left neck dissection Th: thyroid gland; Tr: trachea; RLN: recurrent laryngeal nerve; CCA: common carotid artery; STA: superior thyroid artery; IJV: internal jugular vein; Oe: oesophagus; ITV: inferior thyroid vein (Note: The STA is a rare anatomical variant supplying both upper and lower poles of the thyroid gland). Image captured by the author during anatomical dissection at the University of Leeds

**Figure 2:**
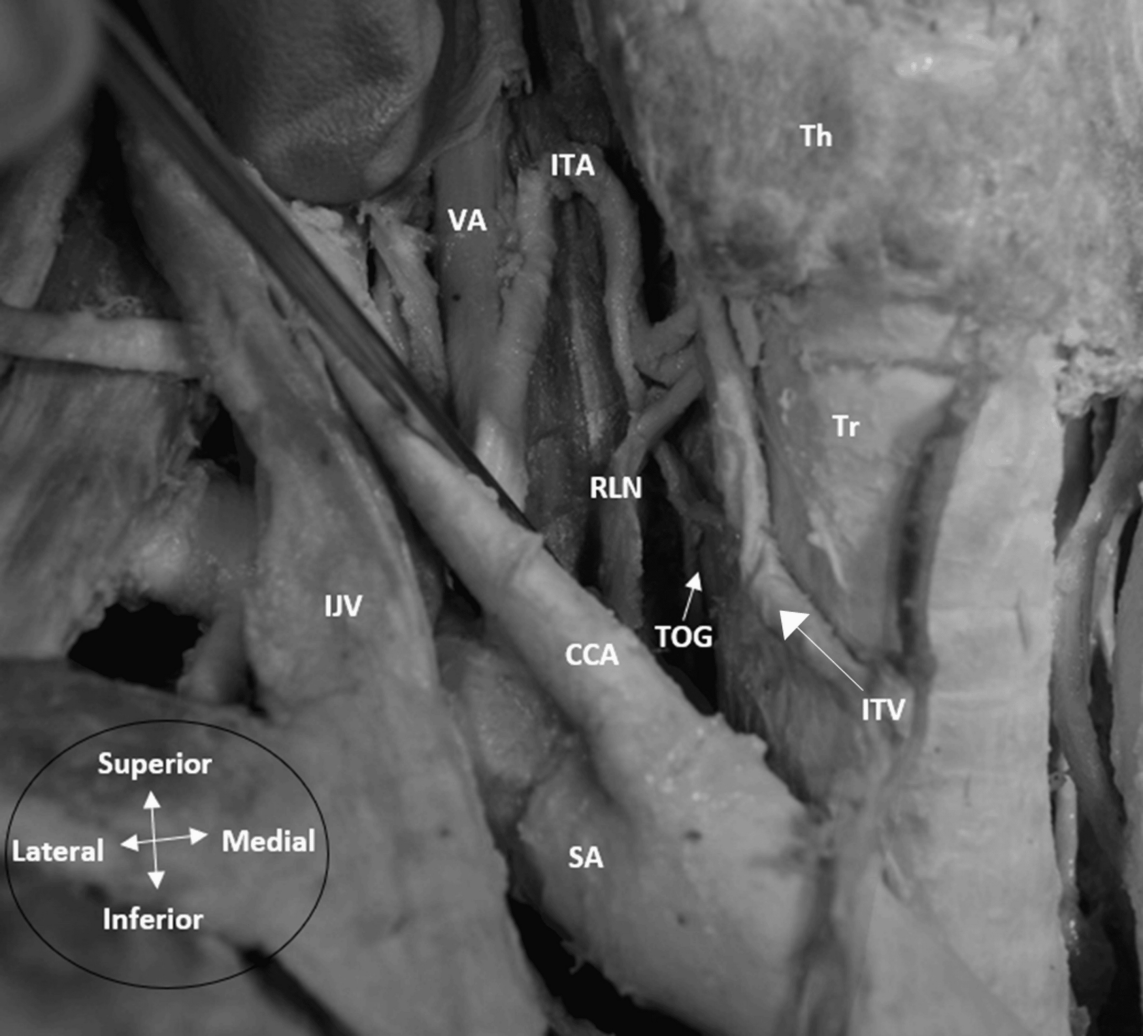
Left recurrent laryngeal nerve located anterolateral to the tracheoesophageal groove on a right neck dissection Key: Th = thyroid gland. Tr = trachea, RLN=recurrent laryngeal nerve, CCA = common carotid artery, ITA=inferior thyroid artery, IJV= internal jugular vein, SA = subclavian artery, VA = vertebral artery, ITV = Inferior thyroid vein. Image captured by the author during anatomical dissection at University of Leeds

Shao et al. mentioned that the RLN lies in the TOG more frequently on the left side than the right, in agreement with several studies [26]. Interestingly, this was also noticed in a dissection bilaterally, where the RLN was found in the TOG on the left side, but anterolaterally on the right side (Figures 1-2). Hence, the TOG might have some utility as a landmark on the left side; however, other studies have found no statistically significant difference in the prevalence of the RLN in the TOG between both sides [23,27].

Additionally, within the meta-analysis, Henry et al. compared 10 studies to assess the prevalence of RLN and its position relative to the TOG. The RLN was located anterior to the TOG in 45.7% of studies, although this was not statistically significant [23,28]. Typically, studies included within the meta-analysis displayed a lack of uniformity in reporting the RLN location in relation to the TOG; hence, a high likelihood of publication bias can be assumed with a lack of quality assessment [28].

At the lower pole of the thyroid, the RLN is commonly accessed via the inferior approach utilising the TOG, and if the nerve is located at a lateral or anterolateral position, there is an increased risk of damaging the RLN when ligating the inferior thyroid vein [29,30]. Furthermore, devascularization of the parathyroid glands is an additional risk due to excessive dissection in the perineural region, causing vascular damage to the parathyroids due to their close proximity to the nerve [31]. Overall, while the TOG may provide some value as a landmark if the nerve is located within it, it is generally considered an inconsistent landmark to use during thyroidectomy, particularly when it is located anteriorly or laterally [28].

Berry’s Ligament

BL is a suspensory ligament consisting of a condensation of pretracheal fascia anchoring the thyroid gland to the laryngotracheal complex [27,32]. Some studies have shown that BL is a reliable landmark in thyroidectomy when assessing the RLN using the superior approach [27,33-35]. However, though the RLN is assumed to be lateral to the BL, some controversy exists as to the exact relationship between the RLN and BL according to other studies [29,32,33]. Nine studies, including one meta-analysis, several case series of cadaveric dissection, and systematic reviews, were analysed.

There are three common relationships shown between the RLN and the BL: superficial, deep, and piercing [28]. A meta-analysis of 16 studies found the nerve superficial to the BL in 78.2% of cases, while it was deep in 14.8% of cases, and only 7% were piercing the ligament [28]. Three studies identified a symmetrical relationship in around three-quarters of cases. Furthermore, when the RLN was superficial to the BL, this arrangement occurred bilaterally in 80.6% of cases [28]. Reeve and Thompson stated that the RLN traverses through BL layers, while Hunt et al. reported that half of the RLNs pierce the BL, in stark contrast to other studies [34-39]. The disparity in the RLN/BL relationship could be explained by the fact that two fascial layers compose the BL: a superficial vascular layer and a deep avascular layer, the true BL [40]. The studies may not have accounted for these two layers when reporting the prevalence of the relationship, potentially underestimating the incidence of the superficial RLN/BL relationship.

There are some risks to the RLN when using the BL as a landmark. Loose areolar tissue attaches both structures, which can confuse an unwary surgeon and precipitate nerve damage [28,40]. Commonly, this is caused by either glandular traction or haemostatic stitches enclosing the nerve when BL remnants are sutured [29]. Bleeding is also a known risk due to thin veins rupturing in the region. In summary, awareness of the layers and meticulous dissection of the true BL could reduce the risk of injury to the RLN [32]. BL is considered a reliable landmark bilaterally, especially for large substernal goitres approached superiorly, since there is a high prevalence of the RLN located superficial to the BL [28,41].

Zuckerkandl Tubercle (ZT)

The ZT is a lateral protuberance of thyroid tissue emerging from the posterior part of the thyroid gland, derived from an embryological remnant of lateral thyroid processes fusing with an ultimobranchial body [42]. The RLN is thought to be posteromedial to the ZT in most cases, but can be located anterior or lateral [43]. The ZT allows the identification of the RLN easily during surgery, acting as an arrow by pointing to the RLN.

Fifteen papers looking at the prevalence and the relationship of the RLN to the ZT were analysed, including a meta-analysis, cadaveric case series, and systematic reviews. The prevalence of ZT is variably reported in the literature, with a meta-analysis by Henry et al. highlighting a 70.2% prevalence in the general population. Various studies have reported the incidence as low as 39%, while others have found the ZT as high as 90.5% [42-45]. Some studies found the incidence of ZT to be higher on the right side, with a prevalence of 83% compared to 52% on the left side [46,47]. These differences in incidence are explained by hypertrophic nodules enlarging the tubercle and a potential lack of awareness by some surgeons identifying the ZT, since its description in anatomical literature is inconsistent [48]. Rajapaksha et al. stated that the right side should be dissected first due to the likelihood of the presence of ZT, which would provide more space to visualize structures on the contralateral side [45]. Although Yun et al. disputed the right-sided prevalence, reporting bilateral ZT in around 91% of cases [49].

In terms of the relation of the RLN to the ZT, a meta-analysis by Henry et al. comparing nine studies showed that the RLN is located mostly posteromedial to the ZT at a prevalence of 82.7%, while it is anterior in 8.6% and lateral in 8.7% [50]. Irawati et al. concluded that because of this posteromedial relationship, present in 90.5% of patients, the RLN was identified in every case by mobilizing the medial portion of the ZT and no subsequent RLNP was detected [44]. The ZT can assist in protecting against RLNP due to its consistent relationship with the RLN when approaching via a superior approach [51]. However, the less common anterolateral or superficial relationships pose the greatest risk due to exposure, and surgeons should be cautious by avoiding the use of electrocautery [52,53].

Some of the potential disadvantages of using the ZT as a landmark include the risk of damaging vessels supplying the parathyroid gland during dissection in its vicinity, which could lead to hypoparathyroidism and hypocalcaemia [54]. Additionally, branching of the RLN can predispose to nerve injury during dissection and may be a factor in the presence of ZT [47]. Furthermore, a pathologically enlarged thyroid gland, such as a goitre, could replicate a ZT, displace the RLN, and increase the risk of RLNP [50]. However, no causal link has been satisfactorily established despite demonstrable statistical significance in the correlation between the size of ZT and the development of complications [54]. In summary, the ZT is a highly valuable landmark due to its high prevalence and consistent relationship with the RLN when present.

Inferior Thyroid Artery (ITA)

The RLN/ITA relationship is highly variable, but is still thought to be a valuable anatomical landmark to avoid injury to the RLN [55]. Approximately over 20 different types of relationships have been identified [56]. The most common are: anterior, posterior, and ITA intertwining with the RLN. Two meta-analyses alongside 14 other articles, including literature reviews, cadaveric studies, and intraoperative studies, were reviewed.

Though the relationship of the RLN being posterior to the ITA is assumed to be the most common, the prevalence of variations can vary enormously among patients undergoing bilateral thyroid surgery, as shown in Table 1. This may give surgeons a false sense of security and increase the likelihood of RLNP. A meta-analysis by Henry et al. compared 79 studies and found that the posterior relationship was the most common at around 50.7%, with the anterior and intertwining relationships displaying pooled prevalences of 27.6% and 21.7% each [57]. Asymmetrical relationships were observed in 64.4% of cases in a pooled analysis, and the left side showed a pooled prevalence of 62.6% for the posterior relationship, with the anterior relationship being only 17.2%. This highlights that many studies have shown the posterior position of the RLN relative to the ITA to be the most frequent overall [55,57-59].

The relationship shows high variability on the right side, whereby anterior and intertwining relationships seem to be increasingly common [55]. The meta-analysis by Henry et al. highlighted that on the right side, there was almost equal prevalence for the posterior and anterior relationships at 37.0% vs 37.1% [57]. The RLN is at the greatest risk in the anterior position or when it intertwines with the ITA [60]. The thyroid gland being mobilised anteriorly can cause stretching of the RLN when the RLN is anterior to the ITA [57]. When the RLN is intertwined, careless ligation of the ITA may cause injury since the RLN may be confused as a branch of the ITA [57, 61-62]. Furthermore, another meta-analysis by Ling and Smoll comparing 32 studies found the posterior relationship to be 50.95% and the anterior and intertwining relationships to be 20.95% and 28.10% respectively, and displayed a posterior relationship in 66.62% of cases [63].

**Table 1:** Rates of RLN/ITA relationship between left and right sides across different studies Both the left side and right side have a higher percentage of the RLN being posterior according to some studies, but some studies suggest anterior or intertwining relationships are more common [27, 47, 59, 63-64]. RLN: recurrent laryngeal nerve; ITA: inferior thyroid artery

Studies	Right (%)			Left (%)		
	Anterior	Intertwined	Posterior	Anterior	Intertwined	Posterior
Matubis et al. [64]	37.0	16.7	46.3	22.2	5.6	72.2
Zada et al. [58]	42.4	9.6	24.4	38.7	5.2	26.6
Uen et al. [27]	20	18.4	61.6	8.3	70.0	21.7
Wojtczak et al. [47]	19.17	3.33	76.6	24.19	0	75.81
Ling and Smoll [63]	34.5	25.5	40.0	23.6	56	56.4
Yalcin [59]	25.5	36.5	23.0	13.7	25.4	52.1

According to Yalcin et al., the use of the ITA as a landmark is not recommended due to the relatively high prevalence of the intertwining position, particularly near the point where they both penetrate the larynx [59]. Cauterisation of ITA branches or application of forceps increases the risk of injury [59]. Furthermore, the ITA is not observed in some cases, reducing its reliability as a landmark; however, another study showed this was only evident in 1-6% of cases [59,65]. Toniato and Boschin disputed this and argued that the ITA is a reliable landmark since the artery can be encircled medially with a vessel loop, and traction applied on the artery can tense the RLN, allowing easy identification [66].

The variability of the RLN/ITA relationship can be complicated by factors such as the branching of the nerve and artery. A meta-analysis by Ling et al. suggested that demographic factors such as gender and race could account for these variations; however, many studies have not analysed these factors [63]. Therefore, selection bias in studies not accounting for demographic factors may play a role in the prevalence of these variations [63]. Surgeons need to factor in these variations when planning and conducting thyroidectomies to avoid RLN injury, particularly when approaching the right side [61]. The ITA may play a partial role as a landmark to prevent RLNP, particularly when the lateral approach is used on the left side [27]. However, its inconsistent variations suggest reduced reliability as a landmark compared to the ZT and BL.

Extralaryngeal Branching (ELB)

ELB is defined as branching of the RLN before the laryngeal entry point and is commonly found near the BL. It is arguably the most important anatomical variation for surgeons to be aware of due to its association with RLNP. Bifurcation, trifurcation, and multiple (≥3) branching are the common patterns noted [67].

ELB is a common variant found in up to 60% of cases, and in 36.5% of individuals it is found bilaterally [68-70]. A meta-analysis by Henry et al. highlighted significant disparity between cadaveric studies, with almost 73% (95% CI: 61.0-84.0) of cases displaying ELB compared to intraoperative findings, where only 39% (95% CI: 29.0-49.0) of cases exhibited ELB [71]. This disparity could be explained by several factors. Intraoperatively, many surgeons do not dissect extensively around BL, leading to proximal branches going unnoticed [72]. Surgeons may find it impractical to search for many small branches due to local inflammation and oedema during thyroidectomy, and hence may not systematically investigate prevalence, leading to underreporting [73,74]. Moreover, ELB was only classified in some intraoperative studies when the RLN branched within 2 cm of the cricothyroid joint [71,75,76]. Other causes for variation between studies could include surgical positioning of the patient (e.g., use of a thyroid pillow can stretch the nerve when extending the neck) [77]. Racial factors may also play a role, with African-Americans having a higher rate of ELB compared to Caucasians, and female gender also being associated with higher rates [72,76,77].

Bifurcation is the most common variant, with a pooled prevalence of 51.1%, followed by no branching [77]. The RLN usually splits into an anterior and posterior branch [70]. Many studies have shown that the anterior branch carries motor supply to the laryngeal muscles, whereas the posterior branch carries sensory information to the mucosa inferior to the larynx [77-79]. The anterior motor branch is at significant risk if bifurcation is not recognised, since the posterior branch may be larger in diameter and assumed to be the sole branch, leading to unintentional transection or traction of the thin anterior branch, subsequently causing RLNP [78,80].

Sancho et al. found a significantly higher rate of RLNP in branched nerves at 15.8% compared to non-branched nerves at 8.1%, with a 2.2 times higher risk of vocal cord palsy (VCP) [81]. Transient rates of VCP were noted to be higher in branched nerves than in unbranched nerves by Barczynski et al., with almost three times greater risk [75]. Other studies have also reported an increased association of RLNP with branching [78,82]. However, these studies differed in how they classified RLNP postoperatively, ranging from 6 months to one year [81,83]. The location of ELB along the cervical segment is important for surgeons to keep in mind to prevent RLNP, since bifurcation can occur at different points. One study revealed 50% of nerves bifurcating after the RLN/ITA crossing, potentially complicating identification [84]. Lastly, Gurleyik mentioned that finding ELB bilaterally can increase the risk of RLN injury, even for very experienced surgeons [85].

Overall, ELB has been associated with RLNP, and the RLN needs to be cautiously approached in the region of the BL, requiring exposure of all branches to reduce the risk of injury.

Non-recurrent Laryngeal Nerve

The non-recurrent laryngeal nerve (NRLN) arises due to a rare embryological anomaly in which the nerve branches directly from the vagus nerve on the right side without looping under the subclavian artery. In almost 90% of cases, this variation is associated with an aberrant right subclavian artery (“arteria lusoria”), originating directly from the aortic arch [86].

Reported prevalence ranges from 0.23% to 4.6%, and a meta-analysis has shown that cadaveric studies report approximately double the incidence compared with intraoperative findings [87]. Intraoperative factors such as oedema, inflammation, and the small calibre of the nerve may contribute to this discrepancy, although cadaveric studies typically involve smaller sample sizes [87]. Nevertheless, the NRLN is often encountered unexpectedly during thyroidectomy without any prior suspicion [88].

The NRLN is highly susceptible to injury during thyroid surgery due to compression, traction, or misidentification, for example, being mistaken for the inferior thyroid artery given its parallel course, which may lead to inadvertent ligature or trauma [89-91]. Some studies report a significantly increased risk of RLNP in association with NRLN, although available data are limited and inconsistent [92].

Clinically, NRLN may be suspected in cases with a right-sided thyroid mass, dysphagia (e.g. dysphagia-lusoria), previous right-sided neck surgery, or abnormal chest imaging suggestive of vascular anomalies such as an aberrant subclavian artery [93]. Preoperative imaging has been shown to reduce the risk of RLNP by up to 75% percent, enabling earlier identification and more efficient dissection of the nerve [94]. Iacobone et al. reported 100% detection of NRLN using preoperative ultrasonography (US), supporting US as a cheap, cost-effective, and non-invasive diagnostic tool [94].

Overall, although the NRLN is rare, its presence confers a substantial risk of RLNP. Preoperative imaging, particularly US, can be incorporated into the preoperative assessment when an NRLN is suspected.

## Conclusions

The RLN is highly susceptible to damage post-thyroidectomy due to its complex anatomy. Though the prevalence of RLNP is highly variable, it can have significant impacts on patients due to several risk factors, with recurrent and malignant goitres being highly associated with injury. Traction, thermal injuries and cautery are the most significant mechanisms of RLNP.

The ZT and BL are the most consistent in their relationship with the RLN and have been found to be the most reliable when identifying the RLN. The RLN is found posteromedially to the ZT in the majority of cases, while the BL is mostly superficial to the RLN. The TOG and the ITA are associated with many different types of relationships to the RLN, particularly on the right side, reducing their reliability as landmarks. The association of RLNP and ELB is significant and has a high incidence, especially on the right side. The risk of injury to the anterior branch, carrying motor innervation to the laryngeal muscles, is high in the region of the BL. While the NRLN may pose a threat during thyroidectomy, its relation to RLN palsy warrants further investigation, and preoperative imaging may be used to mitigate its risk.

Overall, many studies were found to have high levels of heterogeneity, numerous biases such as selection and measurement bias and a lack of statistical significance. Further prospective studies with larger sample sizes should investigate the true clinical impact of anatomical landmarks and variations on RLNP.
